# 6-Gingerol Inhibits Hair Shaft Growth in Cultured Human Hair Follicles and Modulates Hair Growth in Mice

**DOI:** 10.1371/journal.pone.0057226

**Published:** 2013-02-21

**Authors:** Yong Miao, Yabin Sun, Wenjun Wang, Benjun Du, Shun-e Xiao, Yijue Hu, Zhiqi Hu

**Affiliations:** 1 Department of Plastic and Aesthetic Surgery, Nanfang Hospital, Southern Medical University, Guangzhou, Guangdong, China; 2 GCP Office, Nanfang Hospital, Southern Medical University, Guangzhou, Guangdong, China; 3 Cancer Research Institute, Southern Medical University, Guangzhou, Guangdong, China; MGH, MMS, United States of America

## Abstract

Ginger (*Zingiber officinale*) has been traditionally used to check hair loss and stimulate hair growth in East Asia. Several companies produce shampoo containing an extract of ginger claimed to have anti-hair loss and hair growth promotion properties. However, there is no scientific evidence to back up these claims. This study was undertaken to measure 6-gingerol, the main active component of ginger, on hair shaft elongation *in vitro* and hair growth *in vivo*, and to investigate its effect on human dermal papilla cells (DPCs) *in vivo* and *in vitro.* 6-Gingerol suppressed hair growth in hair follicles in culture and the proliferation of cultured DPCs. The growth inhibition of DPCs by 6-gingerol *in vitro* may reflect a decrease in the Bcl-2/Bax ratio. Similar results were obtained *in vivo*. The results of this study showed that 6-gingerol does not have the ability to promote hair growth, on the contrary, can suppress human hair growth via its inhibitory and pro-apoptotic effects on DPCs *in vitro*, and can cause prolongation of telogen phase *in vivo*. Thus, 6-gingerol rather than being a hair growth stimulating drug, it is a potential hair growth suppressive drug; i.e. for hair removal.

## Introduction

Hair is a protective appendage on the body that is considered an accessory structure of the integument. Although the perturbation and loss of hair follicles and alterations in hair fiber production in humans are generally not life-threatening events, their profound impact on social interactions and on patients' psychological well-being is undeniable [1], [2] which in turn increases the demands for the possible treatment options and turns hair loss treatment products into a multi-million dollar industry. Despite this, most current products are ineffective, as evidenced by the fact that the FDA has approved only two treatments for hair loss [3]. Given the limited, transient and somewhat unpredictable efficacy of the current approved anti-hair loss medicines [3], novel pharmacological treatments and agents are on demand. There have been many attempts to discover materials from traditional herbal medicines that prevent hair loss [4]–[8]. For instance, green tea, the tuber fleeceflower (*Polygonum multiflorum* Thunb) and ginger (*Zingiber officinale*) have been traditionally used in East Asia to treat patients suffering from baldness and hair loss. Several companies produce shampoos containing an extract of *P. multiflorum* or ginger extracts which are claimed to have anti-hair loss and hair growth promotion properties [9], [10]. Recently, several studies demonstrated that the green tea [4] or *P. multiflorum* extract [8] can promote hair growth; however, there is no scientific evidence to support this claim for ginger.

The mammalian hair follicle contains dermal papilla (DP),which mainly consist of DP cells (DPCs), and dermal sheath derived from the mesenchyme as well as epithelial cells of the outer root sheath, inner root sheath, matrix and hair shaft, derived from the epithelium [11]. The postnatal hair follicle undergoes a cycle of anagen (growth phase), catagen (regression phase) and telogen (resting phase). The reciprocal interactions between the epithelium and mesenchyme are essential for postnatal hair growth and cycling of hair follicles. The DP is known to have a key role in the regulation of hair growth and cycling [11].Thus, any factor that affects the functions of DPCs can influence hair growth. For instance, Minoxidil [12] and epigallocatechin-3-gallate [4] stimulate the hair growth by exerting their anti-apoptotic effects on DPCs (through increasing the Bcl-2/Bax ratio), this is one of the mechanism of actions on Minoxidil. Cisplatin [13] on the other hands leads to hair loss by inducing apoptotic effects on DPCs (through decreasing the Bcl-2/Bax ratio).

There are several recent scientific investigations aimed at the isolation and identification of active constituents of ginger and the scientific verification of its pharmacological actions and those of its constituents [14], [15]. The results show that 6-gingerol is the most abundant active constituent and has various pharmacological and microbiological effects, including anti-tumorigenic, anti-inflammatory, anti-oxidant and anti-emetic actions; However, pharmacological effects of 6-gingerol on hair growth are not scientifically proven.

In this study, we have investigated the effects of 6-gingerol on human DPCs and hair shaft growth ex vivo and hair growth in mice.

## Materials and Methods

### 2.1. Isolation and culture of human hair follicles

Punch biopsy (4 mm) specimens were taken from male nonbalding occipital scalps of patients undergoing hair transplantation surgery for androgenic alopecia. The Medical Ethical Committee of the Southern Medical University approved all studies described here. The study was conducted according to the Declaration of Helsinki Principles and informed written consent was obtained from all patients. Hair follicles were isolated and cultured as described by Philpott et al [16]. Briefly, hair follicles were isolated with forceps under a binocular light microscope and cultured in 24-well dishes for 7 days in Williams E medium (Gibco BRL, Gaithersburg, MD) supplemented with 10 ng/ml hydrocortisone, 10 mg/ml insulin, 2 mM L-glutamine, 100 U/ml streptomycin and 10 ng/ml hydrocortisone at 37 °C in a 5% (v/v) CO_2_ atmosphere. 6-Gingerol (>95% purity; Sigma Chemical Co., St. Louis, MO) was dissolved in dimethylsulfoxide (DMSO; Solarbio Science & Technology Co., Ltd., Beijing, China) and to keep final concentration of the vehicle < 0.1%. 6-Gingerol was added to culture medium at 10 or 20 µg/ml, and 0.1% (v/v) DMSO was used as a control. In all experiments, culture medium and 6-gingerol were refreshed every other day. A total of 180 anagen hair follicles from 3 different volunteers (60 follicles/subject) were cultured under each growth condition. The mean length of hair follicle from the bottom of the dermal papillae at day zero was 4.5 mm.

### 2.2. Culture of human DPCs

DPCs were isolated and cultured as described by Magerl et al [17]. Briefly, dermal papillae were microdissected from the bulbs of dissected hair follicles, transferred onto plastic dishes and cultured in Dulbecco's modified Eagle medium (DMEM; Gibco, Grand Island, NY) supplemented with 100 U/ml penicillin, 100 mg/ml streptomycin and 20% (v/v) fetal bovine serum (FBS; Gibco) at 37 °C in a humidified 5% CO_2_ atmosphere. The explants were kept in the medium for 7 days and the medium was changed every 3 days. Once cell outgrowth was sub-confluent, cells were harvested with 0.25% (w/v) trypsin-EDTA (Invitrogen, Carlsbad, CA) and subcultured with a split ratio of 1:3. Afterwards, DPCs were maintained in DMEM supplemented with 10% FBS.

### 2.3 Cell proliferation and apoptosis assays

Cell proliferation was determined using the MTT assay as described by Mosmann [18]. Briefly, DPCs (3 × 10^4^ cells/well) were seeded into 96-well plates and incubated for 24 h before adding 6-gingerol at 5 or 10 µg/ml and then incubated at 37 °C for 48 h, using 0.1% DMSO as a negative control. Absorbance at 490 nm was measured using an ELISA reader. Relative cell viability was calculated by dividing the absorbance of treated cells by that of the nontreated cells in each experiment.

DPCs seeded in 6-well plates at a density of 2.5 × 10^5^ cells/well were treated with 5 or 10 µg/ml 6-gingerol or 0.1% DMSO as a control for 48 h. Cell apoptosis was examined using an Annexin V detection kit (Caltag-Medsystems Ltd., Buckingham, UK) according to the manufacturer's instructions. Data acquisition and analysis were done in a FACSort Cytometer (FAC-SCA, New York, NY). A minimum of 2.5 × 10^5^ cell events was acquired for each group in the process of analysis and each experiment was repeated at least 3 times.

### 2.4 Western blot analysis

Both control and 6-gingerol-treated DPCs were collected at 48 h after treatment. Total cell lysates were prepared and 30 µg of protein was subjected to sodium dodecyl sulfate/polyacrylamide gel electrophoresis (SDS-PAGE) followed by immunoblot analysis. Primary antibodies were incubated at appropriate dilutions: anti-Bcl-2 monoclonal antibody, 1:500; anti-Bax monoclonal antibody, 1:500; and anti-GAPDH monoclonal antibody, 1:1000 (Santa Cruz Biotechnology Inc., Santa Cruz, CA). The immune complexes were detected using a western blotting enhanced chemiluminescence (ECL) kit (Santa Cruz) and quantified using analyst/PC densitometry software (Bio-Rad Laboratories, Hercules, CA).

### 2.5 In vivo analysis of 6-gingerol

A total of 36 female C57BL/6 mice (8 weeks old) were purchased from the Experimental Animal Center of Southern Medical University (Guangzhou, China) and divided randomly into 3 groups (12/group). Anagen was induced by depilation of skin on the back of C57BL/6 mice that were in the telogen phase of the cycle, as described [19]. Briefly, hair was removed by topical application of calcium thioglycolate. From the following day (day 1), 0.2 mL of 0.1 mg/mL or 1 mg/mL 6-gingerol in 75% (v/v) ethanol was applied to the dorsal skin of each animal every day for 10 days. Control animals received the vehicle solution alone. The back of each mouse was observed and photographed every 5 days following depilation. All experiments lasted for 20 days and the mice were then sacrificed. The histology of skin specimens taken from the back of each mouse was examined with hematoxylin and eosin (H&E) staining. All experimental manipulations were done in accordance with the National Institutes of Health Guide for the Care and Use of Laboratory Animals and with the approval of the Experimental Animal Ethical Committee of Southern Medical University.

### 2.6 Statistical analysis

All data are expressed as mean ± SD from three independent experiments. All statistical analysis was done with SPSS 13.0. Differences between experimental groups were evaluated by Student's *t*-test. Statistically significant difference was set at *P*<0.05.

## Results

### 3.1 6-Gingerol significantly inhibits the growth of DPCs and induces cell apoptosis

**Figure 1:**
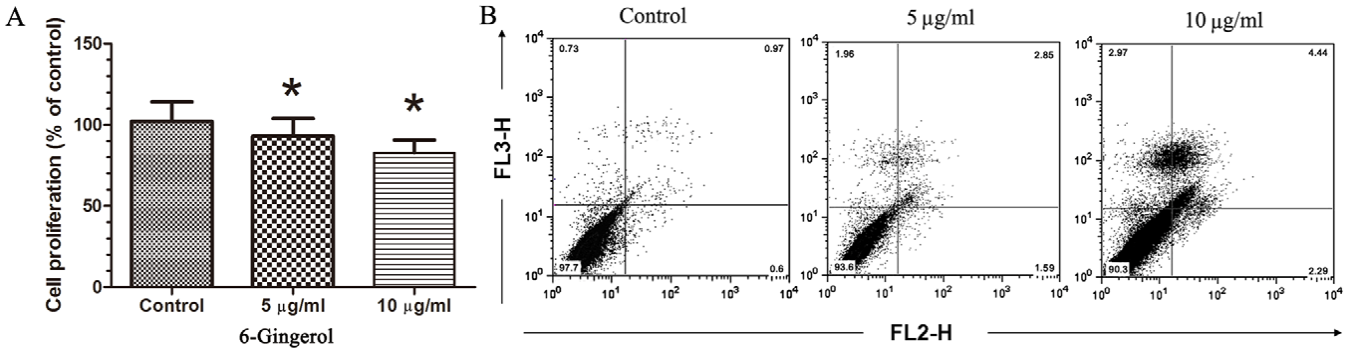
Growth inhibition and induction of apoptosis effects of 6-gingerol on human DPCs *in vitro.* DPCs were cultured with different concentrations of 6-gingerol for 48 h. Compared to the vehicle-treated control, treatment with 10 µg/ml 6-gingerol significantly inhibited the proliferation of cells. (A) Cell viability was determined by the MTT assay. *P<0.05 vs the vehicle-treated control. (B) Apoptosis analysis of cells.

To examine the effect of 6-gingerol on the proliferation of DPCs, we treated DPCs with DMSO (control) or 6-gingerol (5 or 10 µg/ml). Within 48 h of culture, 6-gingerol exhibited a significant dose-dependent growth inhibitory effect on DPCs (Fig. 1A). To determine the mechanisms underlying 6-gingerol-inhibited DPCs growth, the effect of 6-gingerol on cell apoptosis was assessed by staining with fluorescein isothiocyanate (FITC) and Annexin V. Consistently, the exposure of DPCs to 6-gingerol significantly increased cell apoptosis as compared to the control group, suggesting apoptosis as the mechanism underlying 6-gingerol-induced cytotoxicity (Fig. 1B).

### 3.2 Effects of treatment with 6-gingerol on apoptosis regulatory proteins

**Figure 2:**
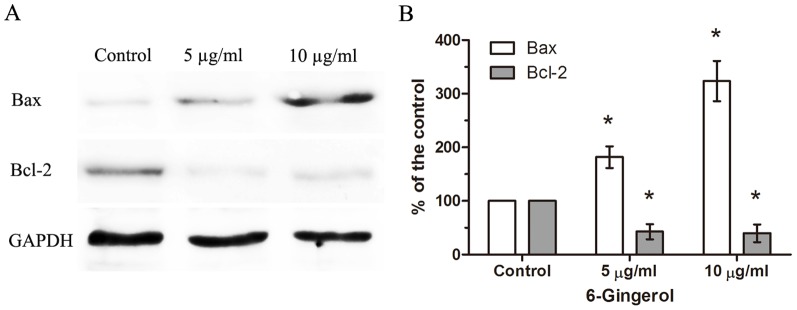
Effect of 6-gingerol on the expression of Bcl-2 and Bax proteins in cultured human DPCs. (A) DPCs were treated with the indicated concentrations of 6-gingerol (0.1% DMSO control) for 48 h and subjected to western blot analysis. Representative bands are shown. (B) Histograms showing the quantification of western blot bands. GAPDH was used as the internal control. *P<0.05 compared to the control (0.1% DMSO) group.

Earlier, 6-gingerol was shown to induce apoptosis through the mitochondrial death pathway [20]–[22]. Because this pathway of apoptosis is known to be regulated by the balance of anti- and pro-apoptotic proteins in the Bcl-2 family [23], [24], we examined the level of expression of two key Bcl-2 family proteins Bcl-2 and Bax in response to treatment of DPCs with 6-gingerol. Immunoblotting studies show that 6-gingerol induced significant down-regulation of the anti-apoptotic Bcl-2 protein and up-regulation of the pro-apoptotic Bax protein compared with the vehicle-treated control. Additionally, Bax protein was up-regulated in a 6-gingerol dose-dependent manner (*P*<0.05) (Fig. 2A, B).

### 3.3 6-Gingerol inhibits hair shaft elongation in cultured human hair follicles

**Figure 3:**
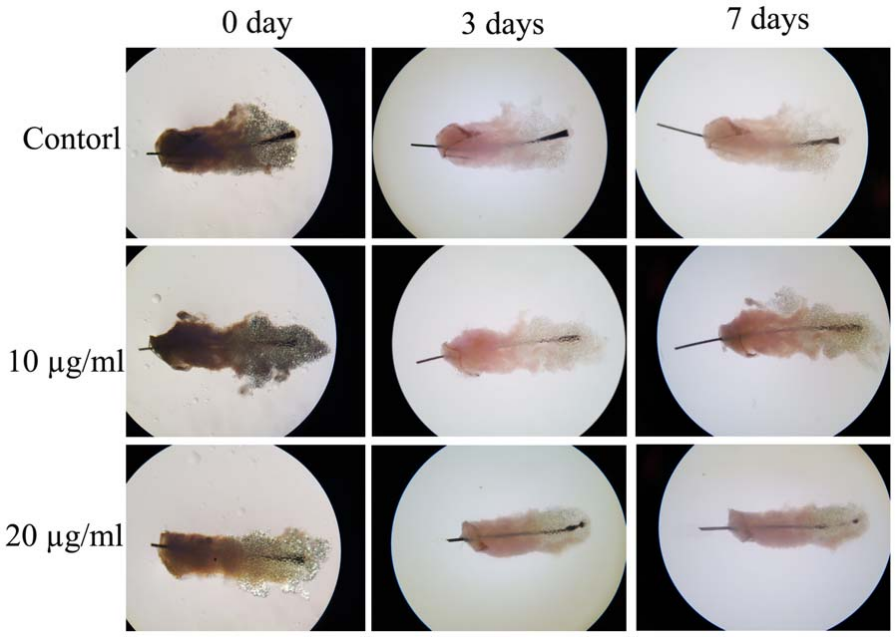
Growth inhibitory effect of 6-gingerol on the elongation of human hair shafts *in vitro.* Hair follicles were treated with different concentrations of 6-gingerol for 7 days and the elongation of human hair shafts was measured using a calibrated eyepiece graticule in a light microscope at a magnification of 20×. (n = 60)

Because DP has a key role in the regulation of hair growth, we investigated the effect of 6-gingerol on hair shaft elongation. Human scalp hair follicles were isolated and cultured both in the absence and in the presence of 6-gingerol. We observed that 20 µg/ml 6-gingerol significantly inhibits elongation of hair shafts (Fig. 3 and Table 1).

**Table 1:** Elongation of hair shafts with the indicated treatment (n = 60).

Treatment	Hair shaft length (mm)	* *P*
Control (0.1% (v/v) DMSO)	1.48 ± 0.20	
6-Gingerol (10 µg/ml)	1.41 ± 0.23	0.11
6-Gingerol (20 µg/ml)	1.06 ± 0.21	0.00

* Versus the control.

### 3.4 6-Gingerol delays anagen induction from telogen in mice

**Figure 4:**
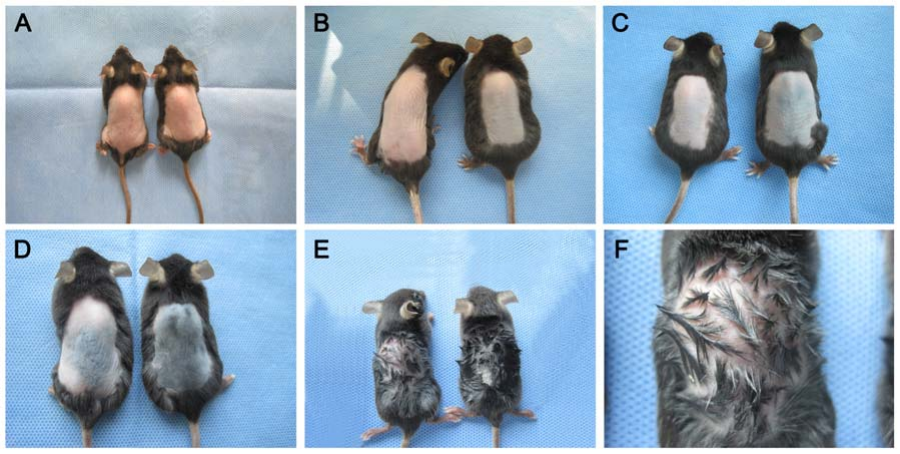
Inhibition of anagen induction from telogen by 6-gingerol in C57BL/6 mice. After depilation, the skin on the back was treated with vehicle (right) or 1 mg/ml 6-gingerol (left) every day for 10 days. Photographs of each animal were taken every 5 days. Compared to the vehicle-treated control, 1 mg/ml 6-gingerol can significantly inhibit anagen induction from telogen. (A) Day zero, (B) 5 days, (C) 10 days, (D) 15 days and (E) 20 days after depilation. (F) Enlarged photograph of the left-hand mouse in (E).

**Figure 5:**
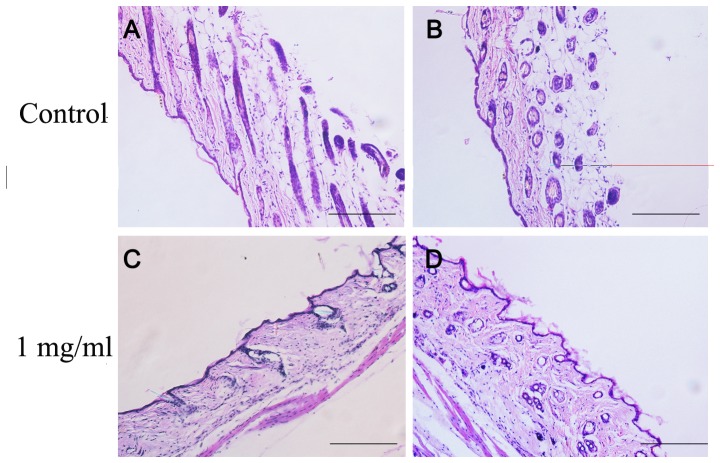
6-Gingerol decreased the number of hair follicles in C57/BL6 mice. After depilation the back, the skin was treated with vehicle alone (control) or 1 mg/ml 6-gingerol every day for 10 days. The effect of 6-gingerol on the hair follicles was analyzed using staining with H&E at 20 days after depilation. (a)–(c) Longitudinal sections of the dorsal skins. (b)–(d) Transverse sections of the dorsal skins. Each scale bar represents 200 µm.

The results given above show only the effect of 6-gingerol in cultured DPCs and hair follicles *in vitro*, which is not the same as the situation in situ. To overcome this limitation, we assessed the role of 6-gingerol in hair growth *in vivo* using 8 weeks old C57BL/6 mice. When the dorsal pelage hair follicles were in the telogen phase, mice were shaved on the back and those showing a uniform telogen stage skin were chosen. The dorsal skin was treated with vehicle alone (control) or with 6-gingerol every day for 10 days. Figure 4 shows hair regrowth at 5-day intervals for the control and for treatment with 6-gingerol (1 mg/ml 6-gingerol as an example). In normal control mice, faint hair regrowth was observed at 10 days after depilation. It became evident that hair on the mice had fully regrown to the original status within ∼20 days. After the topical application of 1 mg/ml 6-gingerol, faint hair regrowth was observed at 15 days after depilation and sparse hair regrowth was observed at 20 days after depilation. The effect of 6-gingerol on the density of hair follicles was further assessed by staining with H&E. Consistently, topical application of 6-gingerol can significantly decrease the number of hair follicles as compared to the control group (Fig. 5).

## Discussion

Ginger (*Z. officinale*) has been traditionally used in East Asia to prevent hair loss and stimulate hair growth. Currently, several companies produce shampoos containing ginger extract and claim that it has anti-hair loss and hair growth promoting effects [9], [10]; however, there is no convincing evidence to support the claimed effects of ginger on hair growth. In this study, 6-gingerol, the main active component of ginger, was found to significantly inhibit hair growth both *in vitro* and *in vivo*. To our knowledge, the present study is the first to evaluate the effect of 6-gingerol on hair growth. Our results show that 6-gingerol does not promote hair growth and can inhibit hair growth by inducing apoptotic effects on DPCs (by decreasing the Bcl-2/Bax ratio).

We found that 6-gingerol in the concentration range 5–10 µg/ml caused a significant dose-dependent inhibition of the proliferation of human DPCs ex vivo. Moreover, 20 µg/ml 6-gingerol significantly suppressed elongation of the hair shaft (1.06 ± 0.21 mm) compared to the control group (1.48 ± 0.20 mm).

6-Gingerol has been shown to induce apoptosis mainly through stimulation of mitochondrial (intrinsic) death pathway [20]–[22], which is regulated primarily by Bcl-2 family proteins, notably the pro-apoptotic Bax and anti-apoptotic Bcl-2 proteins [23], [24]. It is generally accepted that Bcl-2 protects cells from apoptosis and that the activity of Bcl-2 is determined by interaction with Bax [25]. Interestingly, Bcl-2 and Bax have been shown to regulate hair follicle apoptosis during the apoptosis-driven regression phase of the hair cycle (catagen) [26], [27]. These findings have led to the hypothesis that 6-gingerol induces apoptosis in hair follicle cells by regulating the levels of Bcl-2 and Bax. In this study, we showed that 6-gingerol affects the expression of Bcl-2 and Bax in cultured human DPCs. We found 6-gingerol increased Bax and decreased Bcl-2expression in cultured DPCs. Treatment with 6-gingerol for 48 h induced a significant increase in the expression of Bax and decrease in the expression of Bcl-2 compared with the control group. Additionally, Bax protein was up-regulated in a 6-gingerol dose-dependent manner. On the basis of the results of this and earlier research [4], [12], [13], [26], [27], we propose that 6-gingerol induces apoptosis in DPCs by decreasing the level of Bcl-2 and increasing the level of Bax.

We have confirmed that 6-gingerol suppresses hair shaft elongation ex vivo by inducing apoptotic damage in DPCs, but it is not clear whether 6-gingerol can penetrate into hair follicles and cause apoptotic damage in DPCs *in vivo*. Compared to the situation ex vivo, DPCs in hair follicles in situ have a higher resistance to damage factors [28]; thus, to determine whether the changes observed ex vivo occurred *in vivo*, we investigated the hair growth-inhibiting activity of 6-gingerol using 8 weeks old C57BL/6 mice in the stable and consistent telogen phase [19]. The shaved back skin of C57BL/6 mice was treated with daily topical application of 6-gingerol for 10 days. In normal control mice, faint hair regrowth was observed at 10 days and full regrowth was observed at 20 days after depilation. However, in the 1 mg/ml 6-gingerol group, only faint hair regrowth was observed at 15 days and sparse regrowth was observed at 20 days after depilation. To further investigate the hair growth inhibition, we assessed the effect of 6-gingerol on the density of hair follicles by staining with (H&E). Consistently, topical application of 1 mg/ml 6-gingerol can substantially decrease the number of hair follicle as compared to the control group. Thus, it was confirmed that the events initially observed *in vitro* actually occurred *in vivo*.

Unwanted body hair can be emotionally and socially devastating [29], resulting in the search for various treatment modalities. The simplest and most popular method is the use of depilatory materials, which chemically remove hair from the skin surface by dissolving keratin but have only a temporary effect. Unfortunately, there are some side-defects, such us skin allergy, paradoxical hypertrichosis or skin burn [30]. According to the results of this present study, 6-gingerol can decrease the density of hair follicles via inducing apoptotic effects on DPCs, and is a potential permanent hair removal drug.

In summary, we report for the first time that 6-gingerol has no effect on promoting hair growth, on the contrary, can suppress human hair growth via its inhibitory and pro-apoptotic effects on DPCs *in vitro*, and cause prolongation of telogen phase *in vivo*. Thus, 6-gingerol rather than being a hair growth stimulating drug, it is a potential hair growth suppressive drug; i.e. for hair removal. The inadequacies of this study are that this research has slightly fewer cases, the unknown effect of 6-gingerol on follicular epithelial cells and genetic changes of 6-gingerol on dermal papilla cells *in vivo*. Therefore, in future experiments, besides increasing the sample size, we should do further experiments to address the above-mentioned problems.
